# Transcriptomic Analysis of Two *Thioalkalivibrio* Species Under Arsenite Stress Revealed a Potential Candidate Gene for an Alternative Arsenite Oxidation Pathway

**DOI:** 10.3389/fmicb.2019.01514

**Published:** 2019-07-04

**Authors:** Anne-Catherine Ahn, Lucia Cavalca, Milena Colombo, J. Merijn Schuurmans, Dimitry Y. Sorokin, Gerard Muyzer

**Affiliations:** ^1^Microbial Systems Ecology, Department of Freshwater and Marine Ecology, Institute for Biodiversity and Ecosystem Dynamics, University of Amsterdam, Amsterdam, Netherlands; ^2^Department of Food, Environmental and Nutritional Sciences, University of Milan, Milan, Italy; ^3^Research Centre of Biotechnology, Winogradsky Institute of Microbiology, Russian Academy of Sciences, Moscow, Russia; ^4^Department of Biotechnology, Delft University of Technology, Delft, Netherlands

**Keywords:** RNA-Seq, arsenic, resistance, adaptation, sulfur-oxidizing bacteria, soda lake, *soe*ABC

## Abstract

The genus *Thioalkalivibrio* includes haloalkaliphilic chemolithoautotrophic sulfur-oxidizing bacteria isolated from various soda lakes worldwide. Some of these lakes possess in addition to their extreme haloalkaline environment also other harsh conditions, to which *Thioalkalivibrio* needs to adapt. An example is arsenic in soda lakes in eastern California, which is found there in concentrations up to 3000 μM. Arsenic is a widespread element that can be an environmental issue, as it is highly toxic to most organisms. However, resistance mechanisms in the form of detoxification are widespread and some prokaryotes can even use arsenic as an energy source. We first screened the genomes of 76 *Thioalkalivibrio* strains for the presence of known arsenic oxidoreductases and found 15 putative ArxA (arsenite oxidase) and two putative ArrA (arsenate reductase). Subsequently, we studied the resistance to arsenite in detail in *Thioalkalivibrio jannaschii* ALM2^T^, and *Thioalkalivibrio thiocyanoxidans* ARh2^T^ by comparative genomics and by growing them at different arsenite concentrations followed by arsenic species and transcriptomic analysis. *Tv. jannaschii* ALM2^T^, which has been isolated from Mono Lake, an arsenic-rich soda lake, could resist up to 5 mM arsenite, whereas *Tv. thiocyanoxidans* ARh2^T^, which was isolated from a Kenyan soda lake, could only grow up to 0.1 mM arsenite. Interestingly, both species oxidized arsenite to arsenate under aerobic conditions, although *Tv. thiocyanoxidans* ARh2^T^ does not contain any known arsenite oxidases, and in *Tv. jannaschii* ALM2^T^, only *arx*B2 was clearly upregulated. However, we found the expression of a SoeABC-like gene, which we assume might have been involved in arsenite oxidation. Other arsenite stress responses for both strains were the upregulation of the vitamin B_12_ synthesis pathway, which can be linked to antioxidant activity, and the up- and downregulation of different DsrE/F-like genes whose roles are still unclear. Moreover, *Tv. jannaschii* ALM2^T^ induced the *ars* gene operon and the Pst system, and *Tv. thiocanoxidans* ARh2^T^ upregulated the *sox* and *apr* genes as well as different heat shock proteins. Our findings for *Thioalkalivibrio* confirm previously observed adaptations to arsenic, but also provide new insights into the arsenic stress response and the connection between the arsenic and the sulfur cycle.

## Introduction

The genus *Thioalkalivibrio* comprises a group of metabolically diverse, haloalkaliphilic and chemolithoautotrophic sulfur-oxidizing bacteria thriving under extreme conditions in soda lakes. They are part of the family *Ectothiorhodospiraceae* within the Gammaproteobacteria (Sorokin et al., 2001a), and include 10 described species and more than 100 isolated strains (Foti et al., 2006; Sorokin et al., 2012). *In silico* analysis of the genomes of 76 strains classified *Thioalkalivibrio* in 25 genomic species, indicating a high genomic diversity within this genus (Ahn et al., 2017). Concomitantly, members of this genus are able to use different reduced sulfur compounds as electron donors such as sulfide, polysulfide, thiosulfate, polythionates, and elemental sulfur (Sorokin et al., 2001a, 2002a,b, 2003, 2004, 2012; Banciu et al., 2004). Moreover, the strains *Thioalkalivibrio paradoxus* ARh1^T^ (Sorokin et al., 2002b), *Tv.* *thiocyanoxidans* ARh2^T^ (Sorokin et al., 2002b) and *Tv. thiocyanodenitrificans* ARhD1^T^ (Sorokin et al., 2004) are also able to oxidize thiocyanate (Sorokin et al., 2001b; Berben et al., 2017), and *Tv. denitrificans* ALJD^T^ (Sorokin et al., 2001a), *Tv. nitratireducens* ALEN2^T^ (Sorokin et al., 2003), and *Tv. thiocyanodenitrificans* ARhD1^T^ (Sorokin et al., 2004) can also grow anaerobically by denitrification. Recently, Andres and Bertin (2016) and Oremland et al. (2017) detected the presence of an *arx*A gene, which in other bacteria, is responsible for the anaerobic energy-generating oxidation of arsenite [As(III)] to arsenate [As(V)], in the genome of 11 *Thioalkalivibrio* strains. Furthermore, transcripts of the *arx*A gene that were highly similar to genes of *Thioalkalivibrio* were discovered in high abundance in Mono Lake, an arsenic-rich soda lake in eastern California (Edwardson and Hollibaugh, 2017). Soda lakes in this area possess, in addition to their characteristic extreme haloalkaline condition (Jones et al., 1977, 1998), elevated arsenic concentrations that range from 0.8 μM in Crowley Lake, over 200 μM in Mono Lake, to 3000 μM in Searles Lake (Oremland et al., 2004). However, despite the multi-extreme conditions, *Thioalkalivibrio* are found in abundance in these soda lakes (Stamps et al., 2018).

Numerous microorganisms developed mechanisms to detoxify their cells from arsenic and in some cases to even use it as an energy source. Arsenic is well known to be highly toxic to most organisms. It may contaminate soils and groundwaters that are used for food production or as a drinking water source (Mandal and Suzuki, 2002; Cavalca et al., 2013) posing severe threats to human health (Kapaj et al., 2006). The most common forms in the environment are arsenite [As(III)] and arsenate [As(V)] (Smedley and Kinniburgh, 2002), of which the reduced form is more toxic (Hughes, 2002). This toxicity is due to the fact that As(III) is able to deactivate compounds by binding to sulfhydryl groups, as are present in glutathione (Scott et al., 1993) or in cysteines (Shen et al., 2013). As(V), however, can compete with phosphate in biochemical reactions due to its chemically similar structure and properties (Wolfe-Simon et al., 2009; Tawfik and Viola, 2011). To survive the presence of arsenic, prokaryotes can perform detoxification, which includes the reduction of As(V) to As(III) followed by As(III) methylation (Qin et al., 2006) and/or the active export of As(III) out of the cell (Ben Fekih et al., 2018). In the methylation process, the As(III) S-adenosylmethionine methyltransferase ArsM transforms As(III) into methylated As(III) compounds. By this mechanism the cell forms even more toxic, highly volatile organic arsenic compounds that can escape from the cell (Qin et al., 2006). In the active transport system, bacteria pump arsenic out of the cell using the Ars gene system. It first reduces As(V) to As(III) by the arsenate reductase ArsC (Ji and Silver, 1992; Martin et al., 2001) and subsequently pumps the As(III) out by the efflux pump ArsB or ACR3 (arsenic compounds resistance) (Bobrowicz et al., 1997; Wysocki et al., 1997; Meng et al., 2004). The activity of these pumps can be augmented by an ATPase, the ArsA, which increases the resistance to arsenic even more (Rosen et al., 1988; Dey and Rosen, 1995; Rosen, 2002). ArsD is an As(III) chaperone that transfers As(III) to ArsA (Lin et al., 2006, 2007) and it also possesses a weak activity as transacting regulatory protein (Wu and Rosen, 1993). The main transacting regulatory protein of the Ars cluster is ArsR, which functions as a transcriptional repressor that activates transcription in the presence of As(III) (Wu and Rosen, 1991). In addition to detoxification, there are numerous prokaryotes that can generate energy by the oxidation of As(III) using arsenite oxidases Aio (Anderson et al., 1992) or Arx (Zargar et al., 2010), or by the anaerobic reduction of As(V) by the arsenate respiratory reductase Arr (Saltikov and Newman, 2003). These three proteins belong to the dimethyl sulfoxide (DMSO) reductase family of molybdoenzymes, also known as complex iron-sulfur molybdoenzymes (CISM) (McEwan et al., 2002; Rothery et al., 2008). They are composed by a heterodimer of a large subunit (AioA, ArxA, and ArrA) containing the molybdopterin binding site and a small subunit with an iron-sulfur cluster (AioB, ArxB, and ArrB) (Krafft and Macy, 1998; Ellis et al., 2001; Afkar et al., 2003; Zargar et al., 2010). AioC, ArxC, and ArrC are involved in electron transfer and in the case of the ArxC and the ArrC, are transmembrane proteins anchoring the protein to the periplasmic membrane (Stolz et al., 2006; Zargar et al., 2010, 2012; Van Lis et al., 2012; Kalimuthu et al., 2014; Andres and Bertin, 2016; Oremland et al., 2017; Glasser et al., 2018). Only recently, the clade of the Arx arsenite oxidase was discovered in *Alkalilimnicola ehrlichii* MLHE-1^T^ (Hoeft et al., 2007; Richey et al., 2009; Zargar et al., 2010) and in *Ectothiorhodospira* PHS-1 (Zargar et al., 2012), two haloalkaliphilic Gammaproteobacteria isolated from Mono Lake. These bacteria couple oxidation of As(III) as sole electron donor with nitrate reduction (Hoeft et al., 2007; Zargar et al., 2010) or anoxygenic photosynthesis (Kulp et al., 2008; Hernandez-Maldonado et al., 2017), respectively. Interestingly, ArxA is more similar to ArrA than it is to AioA (Richey et al., 2009; Zargar et al., 2010).

The aim of our research was to understand the mechanisms of resistance and adaptation to arsenic within the genus *Thioalkalivibrio*. We first searched in 76 *Thioalkalivibrio* genomes for genes that potentially can be involved in arsenic metabolism. Subsequently, we grew two *Thioalkalivibrio* strains at different As(III) concentrations. For this, we chose *Tv. jannaschii* ALM2^T^, which was isolated from Mono Lake (Sorokin et al., 2002a) where arsenic is present at relatively high concentrations (Oremland et al., 2004), and *Tv. thiocyanoxidans* ARh2^T^, which was isolated from a Kenyan soda lake (Sorokin et al., 2002b). We measured the As(III) oxidation capacity of the two species and performed RNA-Seq analysis to study their gene expression under arsenite stress. To our knowledge, this is the first transcriptomic work done on the arsenite stress response in chemolithoautotrophic bacteria.

## Materials and Methods

### Strains and Growth Conditions

Axenic cultures of *Tv. jannaschii* ALM2^T^ and *Tv. thiocyanoxidans* ARh2^T^ were grown in 200 ml batch cultures at 30°C on a shaker set at 100 rpm. The medium was composed of 17.5 g/l Na_2_CO_3_, 13.9 g/l NaHCO_3_, 6.1 g/l NaCl, 1 g/l K_2_HPO_4_, 0.2 g/l MgCl_2_, 40 mM Na_2_S_2_O_3_, 5 mM NH_4_Cl, and 1:1000 trace metals (Pfennig and Lippert, 1966). Sterile solutions of MgCl_2_, Na_2_S_2_O_3_ and trace elements were added from concentrated stock solutions after autoclaving. The final pH of the culture medium was adjusted to pH 9.8. As(III) as sodium arsenite (NaAsO_2_) (Sigma Aldrich, United States) was added to the medium just before inoculation of the bacteria. For the growth curves of *Tv. jannaschii* ALM2^T^ and *Tv. thiocyanoxidans* ARh2^T^, the cultures were supplemented with 0.1, 0.5, 5, or 7.5 mM As(III). Cultures without As(III) were used as reference and growth of all cultures was monitored daily by measuring the OD at 600 nm. *Tv. thiocyanoxidans* ARh2^T^ and *Tv. jannaschii* ALM2^T^ grew up to a concentration of 0.1 and 5 mM As(III), respectively (Supplementary Figure 1). Therefore, cultures were prepared at 0.1 mM As(III) for *Tv. thiocyanoxidans* ARh2^T^, and at 0.1 and 5 mM for *Tv. jannaschii* ALM2^T^ to study the As(III) resistance mechanisms by transcriptomics. Again, cultures without As(III) were used as reference and their growth was followed by OD measurements at 600 nm. Samples for arsenic species and transcriptomic analysis were taken in the exponential growth phase at an OD_600_ ∼ 0.1, which corresponded to one [reference; 0 mM As(III)] and two [0.1 mM As(III)] days after inoculation for the *Tv. thiocyanoxidans* ARh2^T^ cultures, and after one [reference; 0 mM As(III)], one [0.1 mM As(III)], and five [5 mM As(III)] days for the *Tv. jannaschii* ALM2^T^ cultures. In addition, sterile culture medium was incubated under the same conditions to check for the possibility of chemical As(III) oxidation. To test the growth with As(III) as sole electron donor, *Tv. thiocyanoxidans* ARh2^T^ was cultivated with 0.1 mM As(III), and *Tv. jannaschii* ALM2^T^ with 0.1 and 2.5 mM As(III) in culture medium prepared as described above with the exception of containing 0.025 g/l MgSO_4_ × 7H_2_O and different Na_2_S_2_O_3_ concentrations depending on the culture (0, 1, 5, 10, and 40 mM). All experiments were done in triplicate.

### Arsenic Speciation by ICP-MS Analysis

Culture supernatant was filtered through a 0.2 μm filter and arsenic species were determined according to Kim et al. (2007). To quantify the total As concentration, 5 ml of the filtrate was acidified prior the analysis with 200 μl of 2% (v/v) HNO_3_. For the determination of inorganic arsenic species As(III) and As(V), 5 ml of the filtrate was added to a Sep-Pak^®^ Plus Acell Plus QMA cartridge (Waters, MA, United States). As(V) remained in the cartridge, whereas As(III) passed through. As(III) was collected and acidified with 200 μl of 2% (v/v) HNO_3._ The As(V) was then washed off the cartridge with 5 ml 0.16 M HNO_3_. Total As, As(III), and As(V) concentrations were measured by ICP-MS (Agilent Technologies, United States). Standard solutions ranging from 0 to 1 mg/l of As were prepared from a sodium arsenite (NaAsO_2_) solution (Sigma Aldrich, United States). All measurements were done in triplicate.

### Comparative Sequence Analysis

The phylogenetic tree of ArxA, ArrA, and AioA was constructed based on a multiple alignment of amino acid sequences, which were selected by a BLASTp analysis of 76 *Thioalkalivibrio* genomes (Ahn et al., 2017) and of reference protein sequences. The selected sequences were aligned with MUSCLE (Edgar, 2004) and the tree was built with the software program MEGA7 (version 7.0.26; Kumar et al., 2016) using the Maximum Likelihood method with 1000 bootstrap replicates, the LG model as substitution model and a discrete gamma distribution (+G) as evolutionary rate differences amongst sites.

The phylogenetic tree of the two SoeA clusters was also built with aligned amino acid sequences found in 76 *Thioalkalivibrio* genomes and references, which were selected based on a previous BLASTp analysis. The alignment and the tree construction were calculated following the same protocol as described above.

### RNA-Sequencing

The biomass was collected in 50 ml Greiner tubes and immediately placed into a centrifuge that was precooled to 4°C. The cells were pelleted by centrifugation at 7,000 × *g* for 4 min at 4°C. The supernatant was removed until approximately 2 ml, in which the cells were suspended and transferred to a 2 ml Eppendorf tube. The sample was then centrifuged at 15,000 × *g* for 1 min at 4°C. The supernatant was completely removed, and the cell pellet was immediately frozen in liquid nitrogen and stored at -80°C until further processing.

The frozen cell pellets were homogenized with a mortar and a pestle before being resuspended in QIAzol Lysis Reagent (Qiagen, Germany). Total RNA was extracted and purified with the RNeasy kit (Qiagen) following the manufacturer’s instructions. The purification step comprised a DNase treatment using the RNase-free DNase kit (Qiagen). The concentration was quantified with the NanoDrop ND2000 (Thermo Fisher Scientific, United States) and the integrity of the RNA was checked on the 2200 TapeStation with Agilent RNA ScreenTapes (Agilent Technologies, Netherlands). Ribosomal RNA (rRNA) was removed by the Illumina Ribo-Zero rRNA Removal Kit (Bacteria) (Illumina, United States). Bar-coded RNA libraries were prepared using the Ion Total RNA-Seq kit v2 and the Ion Xpress RNA-Seq barcoding kit according to the supplier’s instructions (Thermo Fisher Scientific). Size distribution and yield were measured on the 2200 TapeStation using Agilent D1000 ScreenTapes (Agilent Technologies). Sequencing templates were prepared on the Ion Chef System with the Ion PI Hi-Q Chef kit (Thermo Fisher Scientific). Samples were sequenced on the Ion Proton platform with an Ion PI Chip v3 (Thermo Fisher Scientific) following the supplier’s instructions.

### RNA-Seq Analysis

The genomes of *Tv. thiocyanoxidans* ARh2^T^ (NZ_ARQK00000000.1) (Berben et al., 2015) and *Tv. jannaschii* ALM2^T^ (NZ_ARLZ00000000.1) were previously sequenced and annotated. The reference gene and genome sequences of both strains were obtained from the NCBI RefSeq FTP server. The software program kallisto (Bray et al., 2016) (v0.44.0) was used to create index files for the quantification from those references. The quality of the reads was assessed by FastQC (version 0.11.7) and estimated to be sufficient. Therefore, no trimming or filtering was performed. Pseudo-alignments were generated in kallisto by mapping the reads from the fastq RNA-Seq files against the indexed reference and reads were quantified using 100 bootstrap samples. Subsequently, differential expression analysis was performed with the software program sleuth (Pimentel et al., 2017) (0.30.0) using the Wald test. The complete differential expression values are presented in Supplementary Table 1 and consists of the NCBI locus-tag, the *b*-value (beta-value), the *P*-value, the *q*-value, the raw counts and the annotation by NCBI for each gene. The *b*-value is a biased estimator of the log fold change and is on a natural-log scale (Pimentel et al., 2017).

The sequences were also analyzed with the RNA-Seq analysis module in the software program CLC Genomics Workbench 11.0.1 (QIAGEN). Proton Torrent fastq files were imported and trimmed using the following default settings: (i) removal of low-quality sequences with a limit of 0.05, (ii) removal of ambiguous nucleotides: maximum 2 nucleotides allowed, and (iii) discard reads below a length of 30 nucleotides. Subsequently, the trimmed reads were mapped to the reference genomes. Differential expression data includes the NCBI locus-tag, the max group mean, the log_2_ (fold change), the fold change, the *P*-value, the FDR *P*-value, and the Bonferroni value (Supplementary Table 2).

## Results and Discussion

### Genomic Features of Arsenic Metabolism and Resistance in *Thioalkalivibrio*

**FIGURE 1:**
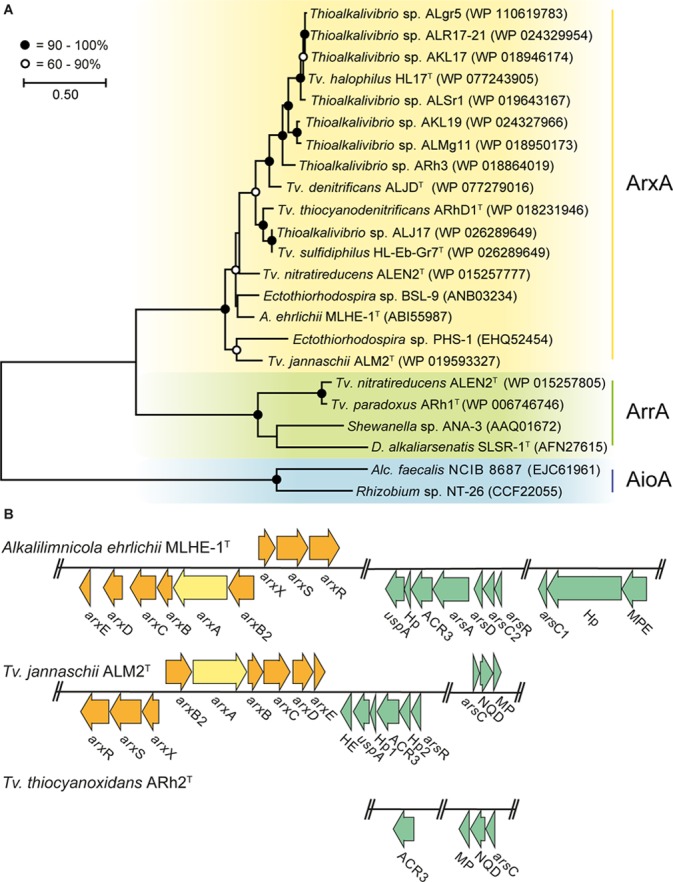
Comparative sequence analysis of arsenic resistance genes in *Thioalkalivibrio*. Yellow, arsenite oxidase ArxA; green, arsenate reductase ArrA; blue, arsenite oxidase AioA; orange, arsenite oxidase *arx* gene cluster; turquoise, arsenic resistance *ars* gene cluster. **(A)** Phylogenetic tree constructed from ArxA, ArrA, and AioA protein sequences present in the genomes of *Thioalkalivibrio* strains and other bacteria. Accession number is provided for each sequence in the figure. **(B)** Arsenic resistance genes in *Alkalilimnicola ehrlichii* MLHE-1^T^, *Tv. jannaschii* ALM2^T^, and *Tv. thiocyanoxidans* ARh2^T^. Hp, hypothetical protein; MPE, metallophosphoesterase; HE, hemerythrin; MP, uncharacterized membrane protein; NQD, NADP(H):quinone dehydrogenase. The locus tags for the genes used in **(B)** are listed in Supplementary Table 3.

We searched in *Thioalkalivibrio* for genes that can be used to grow on arsenic as an energy source. Therefore, a phylogenetic tree was constructed with the putative protein sequences of ArxA, AioA (arsenite oxidases), and ArrA (arsenate reductase) detected in the 76 available genome sequences of different *Thioalkalivibrio* strains (Figure 1A). In those, a putative ArxA was found in 14 *Thioalkalivibrio* strains and a putative ArrA in two. Genes coding for AioA were not detected in any of the strains. *Tv. nitratireducens* ALEN2^T^ was the only strain that contained both ArxA and ArrA. Previously, the presence of ArxA has only been reported in 11 *Thioalkalivibrio* strains (Andres and Bertin, 2016; Oremland et al., 2017) while the presence of ArrA has been never documented. For the strains used in the cultivation experiment, *Tv. jannaschii* ALM2^T^ possesses a putative ArxA while *Tv. thiocyanoxidans* ARh2^T^ lacks any of the known genes to generate energy from inorganic arsenic.

The genomes of *A. ehrlichii* MLHE-1^T^ (another member of the *Ectothiorhodospiraceae*), *Tv. jannaschii* ALM2^T^ and *Tv. thiocyanoxidans* ARh2^T^ encode different gene clusters for the detoxification (*ars* genes) and for the oxidation (*arx* genes) of arsenite (Figure 1B). *A. ehrlichii* MLHE-1^T^ (Zargar et al., 2010, 2012) and *Tv. jannaschii* ALM2^T^ possess an identical *arx* gene cluster for arsenite oxidation and a highly similar set of *ars* genes for arsenic resistance. For the *ars* genes, *A. ehrlichii* MLHE-1^T^ possesses the most complete gene cluster including *ars*ADR, ACR3 and two detoxifying arsenate reductases *ars*C, one glutaredoxin- (*ars*C1) and one thioredoxin-dependent (*ars*C2). In *Tv. jannaschii* ALM2^T^, a more reduced set including an *ars*R, a glutaredoxin-dependent *ars*C and an ACR3 was present. Another annotated ACR3 efflux pump was found in ALM2^T^ outside the shown cluster (Locus-tag: F816_RS0108235) together with three uncharacterized membrane proteins. Interestingly, *A. ehrlichii* MLHE-1^T^ and *Tv. jannaschii* ALM2^T^ also encode for a universal stress protein (*usp*A) in their *ars* gene cluster. On the contrary, *Tv. thiocyanoxidans* ARh2^T^ only possesses a truncated *ars* gene cluster with an ACR3 and a glutaredoxin-dependent *ars*C, also subdivided in two operons, and without *ars*R. Outside of the operon, two putative ArsR for *Tv. thiocyanoxidans* ARh2^T^ were found by BLASTp, but with low identity values, using the ArsR of *A. ehrlichii* MLHE-1^T^ (Locus-tag: Mlg_2713), and of *Tv. jannaschii* ALM2^T^ (Locus-tag: F816_RS0102085) (Supplementary Table 4) as subjects. Neither the genome of *Tv. thiocyanoxidans* ARh2^T^ nor of *Tv. jannaschii* ALM2^T^ contained the *ars*M gene necessary for the detoxification of the intracellular As(III) by methylation (Qin et al., 2006). Furthermore, the genomes of *Tv. jannaschii* ALM2^T^ and *Tv. thiocyanoxidans* ARh2^T^ were screened for the presence of *ars*HIJNOPTX via BLASTp. In *Tv. jannaschii* ALM2^T^, an ArsI [Locus-tag: F816_RS0102080; query cover of 93% and an identity of 41% with ArsI of *Bacillus* sp. MD1 (AIA09488)] was found inside the *ars* gene operon as well as a second putative ArsI positioned directly besides the *ars* gene operon [Locus-tag: F816_RS14315 (78% query cover and 29% identity to AIA09488)]. In addition, putative sequences for ArsJ were detected in *Tv. jannaschii* ALM2^T^ [Locus-tag: F816_RS0106725; query cover of 95% and a similarity of 60.3% with ArsJ of *Pseudomonas aeruginosa* (WP_003109849)] and in *Tv. thiocyanoxidans* ARh2^T^ (Locus-tag: G372_RS0110690; query cover of 94% and an identity of 59,1% with WP_003109849). The putative ArsJ in *Tv. jannaschii* ALM2^T^ is encoded together with an annotated glyceraldehyde 3-phosphate dehydrogenase (GAPDH) in the same operon. The combination of these two genes has been described to confer resistance to As(V) (Chen et al., 2016). However, the GAPDH was not found in the operon of the putative ArsJ in *Tv. thiocyanoxidans* ARh2^T^. To prove the function of these putative ArsIJ, experimental evidence must follow.

### Physiological and Transcriptomic Response to Arsenic Stress

*Tv. jannaschii* ALM2^T^ and *Tv. thiocyanoxidans* ARh2^T^ were cultivated in the presence of different concentrations of As(III) to determine their resistance. *Tv. jannaschii* ALM2^T^ resists much higher As(III) concentrations than *Tv. thiocyanoxidans* ARh2^T^ (Supplementary Figure 1). *Tv. thiocyanoxidans* ARh2^T^ was only able to grow until a concentration of 0.1 mM As(III), whereas *Tv. jannaschii* ALM2^T^ still grew up to 5 mM As(III). All cultures grew aerobically with thiosulfate as an electron donor and they could not grow with As(III) as their sole potential electron donor (Supplementary Figure 2).

To gain deeper insight in their resistance mechanism against arsenic, *Tv. thiocyanoxidans* ARh2^T^ and *Tv. jannaschii* ALM2^T^ were both cultivated in absence of As(III) (reference) and at 0.1 mM As(III). Furthermore, *Tv. jannaschii* ALM2^T^ was also grown at 5 mM As(III). Cultures were harvested in their exponential growth phase to measure the arsenic species composition in the culture fluid and to determine gene expression in both *Thioalkalivibrio* species.

**FIGURE 2:**
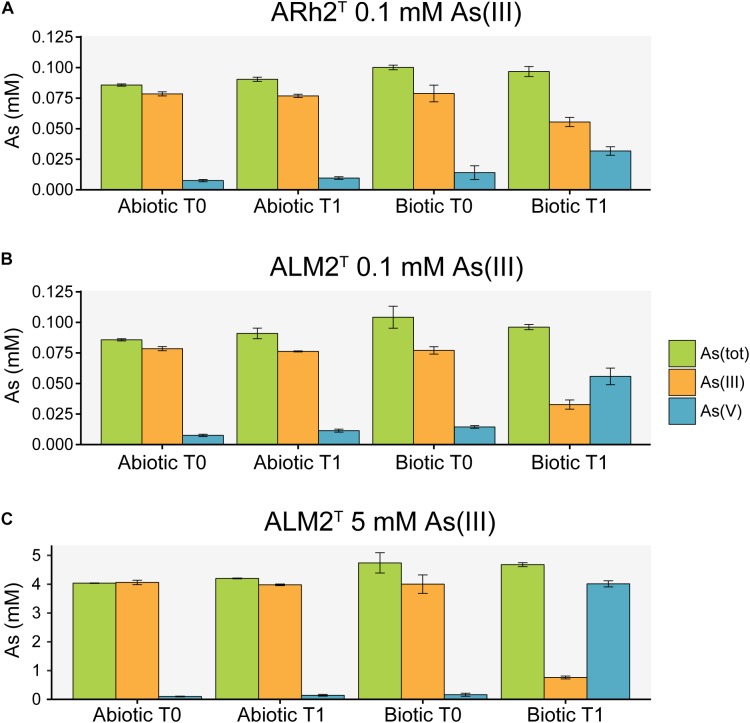
Arsenic species analysis of culture fluid from sterile samples (Abiotic) and samples inoculated with *Tv. thiocyanoxidans* ARh2^T^ and *Tv. jannaschii* ALM2^T^ (Biotic) at the inoculation (T0) and sampling time (T1). **(A)** ARh2^T^ culture incubated with 0.1 mM As(III) for 2 days. **(B)** ALM2^T^ culture incubated with 0.1 mM As(III) for 1 day. **(C)** ALM2^T^ culture incubated with 5 mM As(III) for 5 days.

Arsenic species were measured in the culture medium at the beginning and at the end of the experiment to investigate the potential of both strains to oxidize As(III) to As(V) under aerobic conditions (Figure 2). Additional sterile samples were analyzed to determine the possibility of chemical oxidation of As(III) in the culture medium. Samples inoculated with *Tv. jannaschii* ALM2^T^ and *Tv. thiocyanoxidans* ARh2^T^ showed a decrease in As(III) and an increase in As(V) over time. During the same incubation time, As(III) and As(V) concentrations did not change significantly in the sterile samples indicating that As(III) oxidation was biologically induced. *Tv. jannaschii* ALM2^T^ had a much stronger As(III) oxidizing capacity as compared to *Tv. thiocyanoxidans* ARh2^T^. When grown in the presence of 0.1 mM As(III), *Tv. jannaschii* ALM2^T^ oxidized 57% of the present As(III) in 1 day, whereas *Tv. thiocyanoxidans* ARh2^T^ only oxidized 26% after 2 days. Most importantly, when grown in the presence of 5 mM As(III), *Tv. jannaschii* ALM2^T^ was able to oxidize 79% of As(III) within 5 days. These findings resemble previous incubation experiments of Mono Lake surface waters that showed a clear link between aerobic As(III) oxidation capacity and added sulfide or thiosulfate (Fisher et al., 2008). In their research, sulfide-amended lake brines showed the formation of thioarsenates compounds from As(III), which were fairly stable in sterile, oxic surface waters, but which were further oxidized to As(V) in samples containing sulfur-oxidizing bacteria. Molecular analysis of the enrichments identified bacteria closely related to *Tv. jannaschii, Tv. versutus* and *Tv. nitratis*. Furthermore, Edwardson et al. (2014) showed that pure cultures of *Tv. jannaschii* ALM2^T^ were able to oxidize monothioarsenate aerobically, but also that they did not show growth with monothioarsenate as their sole electron donor. In our research, we have now demonstrated growth of *Tv. jannaschii* ALM2^T^ and *Tv. thiocyanoxidans* ARh2^T^ with As(III) in combination with thiosulfate. Whether *Thioalkalivibrio* can gain energy from As(III) or thioarsenate oxidation, or that this oxidation is only used for detoxification purposes, remains an open question. However, it can be excluded that these compounds support growth as a sole electron donor.

Transcriptomic analysis enabled screening for key genes in the metabolism of and in the resistance against arsenic, and it shows differences in gene expression between *Tv. jannaschii* ALM2^T^ and *Tv. thiocyanoxidans* ARh2^T^. General information on the individual RNA-seq samples analyzed by kallisto and sleuth are presented in Supplementary Table 5 and the complete expression data can be found in Supplementary Table 1. In total, 57.4 million sequence reads were produced by the Ion Proton platform ranging from 2.3 million to 4.9 million sequence reads per sample. From those reads, between 57.9% and 72.5% could be assigned to an open reading frame (ORF) depending on the sample analyzed with kallisto. In *Tv. jannaschii* ALM2^T^, 2833 ORFs were detected, and 2716 ORFs in *Tv. thiocyanoxidans* ARh2^T^. For the analysis performed with sleuth, an ORF is considered differentially expressed if the *b*-value is greater than 0.7-fold and its *P*-value is lower than 0.1. The RNA-Seq data analyzed by sleuth gave 101 up- and 84 downregulated genes for *Tv. thiocyanoxidans* ARh2^T^ at 0.1 mM As(III) [0.1 mM vs. 0 mM As(III)] (Supplementary Table 6), only two up- and one downregulated genes for *Tv. jannaschii* ALM2^T^ at 0.1 mM As(III) [0.1 mM vs. 0 mM As(III)] (Supplementary Table 7), and 26 up- and 16 downregulated genes for *Tv. jannaschii* ALM2^T^ at 5 mM As(III) [5 mM vs. 0 mM As(III)] (Supplementary Table 8). As certain pathways could not be completely revealed based on the sleuth results only, we decided to also analyze the RNA-Seq data with CLC Genomics Workbench (Supplementary Table 2). With CLC, between 77.74 and 82.2% of the reads could been allocated to an ORF. Here, an ORF was considered to be differentially expressed if the log_2_ fold change was higher than 1-fold and its *P*-value lower than 0.1. With this threshold, CLC found 99 up- and 91 downregulated genes for *Tv. thiocyanoxidans* ARh2^T^ at 0.1 mM As(III) [0.1 mM vs. 0 mM As(III)], four up-, and five downregulated genes for *Tv. jannaschii* ALM2^T^ at 0.1 mM As(III) [0.1 mM vs. 0 mM As(III)], and 40 up- and 20 downregulated genes for *Tv. jannaschii* ALM2^T^ at 5 mM As(III) [5 mM vs. 0 mM As(III)].

The quality of the RNA-Seq data analyzed by kallisto and sleuth was evaluated by principal component analysis and plotted in a graph with the first two principal components as axes (Supplementary Figure 3). On the first principal component, the samples of each condition cluster together and were well separated from the other conditions. Remarkably, the *Tv. jannaschii* ALM2^T^ samples grown at 0, 0.1, and 5 mM As(III) are not ordered based on the increasing As(III) concentration on the first principle component, but in the order of 0.1, 0, and 5 mM As(III). This phenomenon might be explained by a hormesis reaction, in which an agent, here As(III), at lower level exposes an beneficial effect on the organism and becomes only toxic at higher concentrations (Mattson, 2008).

**FIGURE 3:**
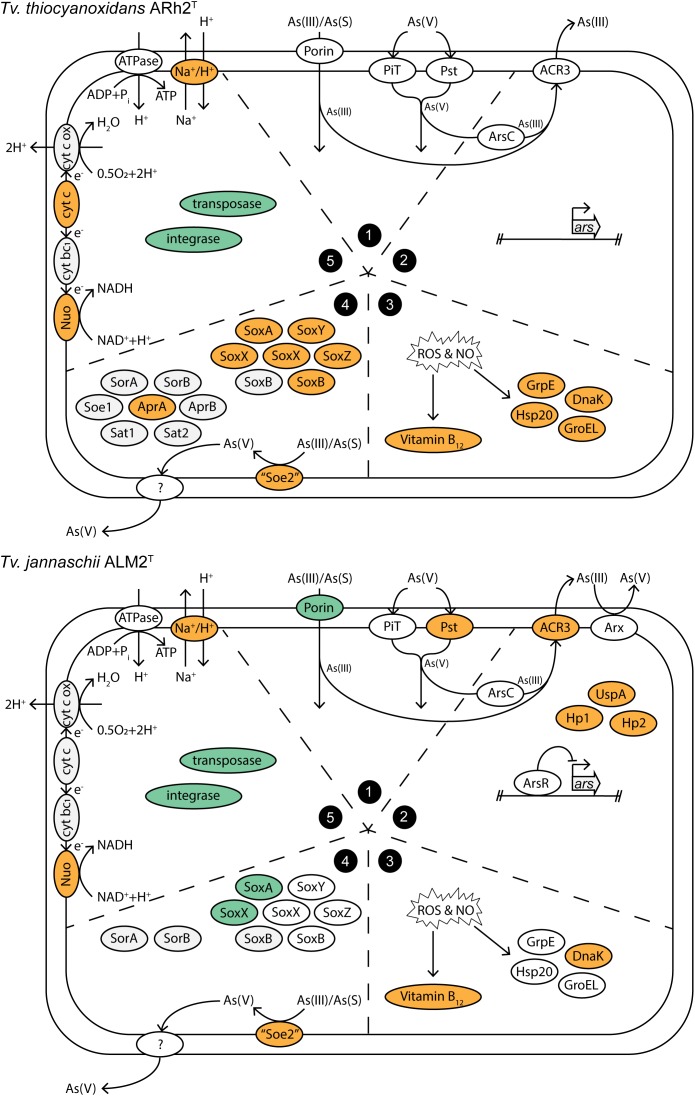
Conceptual model of cellular processes within *Tv. jannaschii* ALM2^T^ and *Tv. thiocyanoxidans* ARh2^T^ under As(III) stress. The section numbers correspond to the subgroups in the Results and Discussion section: (1) Arsenic influx into the cell, (2) Arsenic metabolism and detoxification, (3) Response to oxidative damage by arsenite, (4) Sulfur metabolism, and (5) Recombination and energy generation. Upregulated genes are colored in orange and downregulated genes in turquoise. Cyt bc_1_, cytochrome bc_1_; cyt c, cytochrome c; cyt c ox, cytochrome c oxidase; As(S), thioarsenate; Hp, hypothetical protein. Locus tags and differential expression values are listed in Supplementary Table 9.

We summarized the results of the gene expression under As(III) stress in a conceptual model (Figure 3), in which the groups correspond to the subgroups of the discussion: (1) Arsenic influx into the cell, (2) Arsenic metabolism and detoxification, (3) Response to oxidative damage by arsenite, (4) Sulfur metabolism, and (5) Recombination and energy generation.

### (1) Arsenic Influx Into the Cell

The arsenic species As(III) and As(V) are able to enter the cell by transporters of molecules whose properties they mimic. In *E.coli*, As(III) can enter the cell by the aquaglyceroporin channel GlpF (Sanders et al., 1997; Meng et al., 2004). These channels normally transport small uncharged molecules such as glycerol (Heller et al., 1980; Borgnia and Agre, 2001), but also As(III) as non-charged As(OH)_3_ under neutral pH (Ramírez-Solís et al., 2012). However, in environments with a pH higher than its pKa of 9.2, As(III) will be mostly present in its ionic form (Smedley and Kinniburgh, 2002). This is the case for soda lakes whose pH ranges from 9.5 to 11 and, from which most *Thioalkalivibrio* strains were isolated (Sorokin et al., 2014). Furthermore, thioarsenates are formed in oxic alkaline brines containing sulfide (Stauder et al., 2005; Planer-Friedrich et al., 2007, 2009; Fisher et al., 2008; Härtig and Planer-Friedrich, 2012), conditions present as well in Mono Lake (Hollibaugh et al., 2005). Until now, it is unknown how As(III) or thioarsenates enter the cells under these conditions. A possible porin involved in their influx could be F816_RS0109535 (Supplementary Table 2) in *Tv. jannaschii* ALM2^T^ as it is highly downregulated at 0.1 and 5 mM As(III). However, no similar protein could be detected in the genome of *Tv. thiocyanoxidans* ARh2^T^. The downregulation of a porin responsible for As(III) or thioarsenate influx would keep the intracellular arsenic concentration lower in *Tv. jannaschii* ALM2^T^, thus conferring a higher As(III) resistance to the strain.

As(V) possesses a similar chemical structure and properties as phosphate (Wolfe-Simon et al., 2009), and can therefore be taken up by the phosphate uptake systems Pit (inorganic phosphate transporter) and Pst (specific phosphate transporter) (Rosenberg et al., 1977; Willsky and Malamy, 1980a), of which Pst is more specific for phosphate and transports As(V) less efficiently (Willsky and Malamy, 1980b; Elias et al., 2012). The gene for the Pit transporter system did not significantly change in expression in any of the samples. However, when grown with 0.1 mM As(III), *Tv. jannaschii* ALM2^T^ upregulates the *pst*A contemporary with the formation of 0.056 mM As(V) (Figure 2). In the presence of 5 mM As(III), the *pst*ABCS and the regulator *pho*U were upregulated with the simultaneous occurrence of 4.01 mM As(V) in the culture medium (Figure 2). These genes did not change in expression in *Tv. thiocyanoxidans* ARh2^T^ cultures, which could be explained by the low As(V) concentration of 0.032 mM at the sampling time (Figure 2). Since the Pst transporter is more specific for phosphate than for As(V), many bacteria increase the expression and the production of Pst to increase phosphate uptake (Andres and Bertin, 2016). This would give another advantage for growth in combination with As(III) oxidation for *Tv. jannaschii* ALM2^T^. However, as both strains were able to tolerate 30 mM As(V) (data not shown), it is possible that *Tv. thiocyanoxidans* ARh2^T^ also possesses a similar mechanism, which was not upregulated with the low As(V) concentration deriving from the oxidation process.

### (2) Arsenic Metabolism andDetoxification

Transcriptomic analysis of *Tv. jannaschii* ALM2^T^ grown at 5 mM As(III) showed an upregulation of the arsenite oxidase *arx*B2 gene, but not of the structural component genes *arx*ABC (Supplementary Tables 1, 2). The Arx protein is only known to work under anaerobic condition coupled to denitrification or anaerobic photosynthesis (Hoeft et al., 2007; Kulp et al., 2008; Zargar et al., 2010; Hernandez-Maldonado et al., 2017). Moreover, as already discussed before, no growth was observed in the tested strains under aerobic condition with As(III) as sole electron donor (Supplementary Figure 2). Previously, Arx has been shown *in vitro* to function as a bidirectional enzyme able to oxidize As(III) and to reduce As(V) (Richey et al., 2009). Due to the presence of Arx in *Tv. jannaschii* ALM2^T^ and the incapacity of this strain to perform denitrification, it could be hypothesized that this strain uses Arx to reduce As(V) to As(III) in combination with the oxidation of reduced sulfur compounds under microoxic/anoxic conditions.

Most organisms perform an active extrusion of As(III) as their main arsenic resistance mechanism. This is performed in prokaryotes by the arsenic resistance *ars* operon, where As(III) produced by the arsenate reductase ArsC is pumped out of the cell by ArsB/ACR3 (Ben Fekih et al., 2018). The *ars* genes were not differentially expressed in *Tv. thiocyanoxidans* ARh2^T^ at 0.1 mM As(III) (Supplementary Table 1). For *Tv. jannaschii* ALM2^T^ at 0.1 mM As(III), only the *usp*A gene was upregulated. In contrast, the *ars* gene cluster was highly expressed in *Tv. jannaschii* ALM2^T^ at 5mM As(III) including the ACR3 efflux pump, two hypothetical proteins and the *usp*A gene. An exception was the *ars*C, which explains the high concentration of As(V) observed in the medium at the time of sampling (Figure 2 and Supplementary Tables 1, 2). The *usp*A gene encodes a *usp*A that is known to be induced under different stress situations (Kvint et al., 2003), by which it increases the endurance of the cell (Nyström and Neidhardt, 1994). Upregulation of this gene has been shown in bacteria under As(III) stress (Weiss et al., 2009; Cleiss-Arnold et al., 2010; Sacheti et al., 2013). Finally, an operon, which is located next to the *arx* and *ars* cluster in *Tv. jannaschii* ALM2^T^ and which includes a putatively annotated glycosyl transferase involved in the cell wall biosynthesis and a rhodanese-related sulfurtransferase were highly upregulated at 5mM As(III) in ALM2^T^. However, in *Tv. thiocyanoxidans* ARh2^T^, this operon is neither upregulated nor found next to the *ars* gene cluster.

### (3) Response to Oxidative Damage by Arsenite

Arsenic has been shown to induce formation of reactive oxygen species (ROS) and nitric oxide (NO) inside the cell (Andres and Bertin, 2016; Zhang et al., 2016). These radicals can cause damage to nucleic acids, proteins, and lipids (Flora, 2011; Birben et al., 2012; Ray et al., 2012; Espinosa-Diez et al., 2015). To reduce the oxidative damage by arsenic, bacterial cells have developed various responses including the upregulation of Fe- and Mn- superoxide dismutases, thiol peroxidases, thioredoxin reductases, thioredoxins, glutaredoxins, glutathione, organic hydroperoxide resistance proteins, and vitamin B_6_ (Andres and Bertin, 2016). In our experiments, however, we did not detect changes in expression for the two *Thioalkalivibrio* strains for any of the genes involved in known antioxidation pathways. However, the As(III) concentrations of 0.1 mM As(III) for *Tv. thiocyanoxidans* ARh2^T^ and 5 mM As(III) for *Tv. jannaschii* ALM2^T^ triggered upregulation of the complete vitamin B_12_ (cobalamin) synthesis pathway (Supplementary Tables 1, 2). Vitamin B_12_ has been shown to protect eukaryotic cells from oxidative damage by its antioxidant activity (Birch et al., 2009; Suarez-Moreira et al., 2009; Moreira et al., 2011; Alzoubi et al., 2012; Bito et al., 2017) as well as when it is generated by arsenic in hepatic rat cells (Chattopadhyay et al., 2012; Majumdar et al., 2012). In bacterial cells, vitamin B_12_ has also been shown to be an antioxidant able to protect the cell from oxidative stress in the acidophilic iron-oxidizing bacterium *Leptospirillum* group II CF-1 (Ferrer et al., 2016). Recently, Qin et al. (2018) discovered that the archaea *Nitrosopumilus maritimus* SCM1 produces vitamin B_12_ under Cu^2+^ stress. Moreover, *cob*STU was shown to be expressed by bacteria in an arsenic-rich acid mine drainage, but was only related to the activation of iron oxidation (Bertin et al., 2011). Here, we propose that vitamin B_12_ is the main antioxidant produced under As(III) stress in *Thioalkalivibrio*.

In addition, *Tv*. *thiocyanoxidans* ARh2^T^ significantly upregulates the expression of the chaperones *dna*K and Hsp20, and slightly of *gro*EL, *grp*E and *dna*J. In contrast, *Tv. jannaschii* ALM2^T^ does not change the expression of those genes. Chaperones from the Hsp70 (DnaK, DnaJ and GrpE) and Hsp60 (GroEL and GroES) systems have been shown to be commonly induced as an arsenic stress response in bacteria (Andres and Bertin, 2016). These chaperones are essential for the cell viability and the survival under diverse stressful conditions as they facilitate the proper folding of newly translated proteins or maintain it for already translated ones (Houry, 2001; Hayer-Hartl et al., 2016; Hartl, 2017).

### (4) Sulfur Metabolism

*Thioalkalivibrio* strains are sulfur-oxidizing bacteria that possess a high inter-genus diversity of different genes and pathways involved in sulfur oxidation (Berben et al., 2019). *Tv. thiocyanoxidans* ARh2^T^ and *Tv. jannaschii* ALM2^T^ differentiate from each other by the fact that *Tv. thiocyanoxidans* ARh2^T^ possesses the TcDH pathway for thiocyanate oxidation, the Apr-Sat pathway for sulfite oxidation as well as a second homologous copy of Soe for sulfite oxidation, whereas *Tv. jannaschii* ALM2^T^ does not (Berben et al., 2019). Interestingly, the sequences of the two SoeA in *Tv. thiocyanoxidans* ARh2^T^ form two distinct clusters, i.e., one cluster grouping around SoeA of *Allochromatium vinosum* (cluster 1) (Dahl et al., 2013) and a second cluster forming a separate group (cluster 2), hereafter called “Soe-like” gene. Both copies are present together in 41 *Thioalkalivibrio* strains (Supplementary Figure 4). Various genes in the sulfur oxidation pathway are upregulated in *Tv. thiocyanoxidans* ARh2^T^ under As(III) stress [*sox*YZXXAB, *apr*A and *soe*ABC (cluster 2)], whereas others were not differentially expressed, such as *soe*ABC (cluster 1), *sat* and *sor*AB. In *Tv. jannaschii* ALM2^T^, only the *soe*ABC (cluster 2) was highly upregulated together with genes necessary for the molybdenum cofactor production of SoeA, the *moa*A (GTP 3′,8-cyclase) (Mendel and Leimkühler, 2015) and the molybdate ABC transporter.

As(III) was also oxidized in *Tv. thiocyanoxidans* ARh2^T^ cultures, although this strain does not possess an arsenite oxidase in its genome (Figure 1). Therefore, another as yet unknown enzyme must exist besides the two known arsenite oxidases AioA and ArxA involved in As(III) oxidation under aerobic conditions. Comparing our results with the work of Fisher et al. (2008), we can hypothesize that thioarsenate species have also formed in our cultures, opening new possibilities for enzymatic pathways of As(III) oxidation in the presence of thiosulfate or sulfide. Some enzymes have already been hypothesized to be involved in the oxidation pathway of thioarsenate compounds. Edwardson et al. (2014) proposed the Sox pathway as a potential facilitator of thioarsenate oxidation based on the structural similarity between monothioarsenate and thiosulfate. In this system, Sox enzymes would be able to cleave the thiol group from monothioarsenate. This hypothesis could be supported by the upregulation of the *sox* cluster in *Tv. thiocyanoxidans* ARh2^T^, but is contradicted by the stable or even slight downregulation of these genes in *Tv. jannaschii* ALM2^T^ where the strongest As(III) oxidation occurred. Furthermore, a sulfide:quinone oxidoreductase and its operon were found upregulated in the presence of sulfide or As(III) in *Synechocystis* sp. strain PCC6803 (Nagy et al., 2014). As these genes are closely related to genes in *Tv. thiocyanodenitrificans* ARhD1^T^, although not found in a single operon in this strain, they proposed that these genes could be involved in thioarsenate oxidation in *Thioalkalivibrio*. However, no upregulation of these genes was detected in the dataset obtained with *Tv. jannaschii* ALM2^T^ and *Tv. thiocyanoxidans* ARh2^T^ growing with As(III). Couture et al. (2012) proposed in a review the involvement of the proteins SelD and SelU coupled to ArxC in thioarsenate production alongside to their normal activity of making selenophosphate and modifying RNA. However, we could not find any homologs to these proteins in the genomes of *Tv. jannaschii* ALM2^T^ and *Tv. thiocyanoxidans* ARh2^T^.

The only genes of the sulfur oxidation pathway that were induced in both strains at their highest respective As(III) concentration were the quinone-dependent sulfite oxidase *soe*ABC (cluster 2). SoeABC is a molybdopterin oxidoreductase of the same family as the arsenic oxidoreductases Aio, Arr, and Arx (Krafft and Macy, 1998; Ellis et al., 2001; McEwan et al., 2002; Zargar et al., 2010; Dahl et al., 2013). Similar to those proteins, SoeA and SoeB form a heterodimer, which is anchored to the cytoplasmic membrane by SoeC. The difference between the SoeA and the arsenic oxidoreductase is that SoeA does not contain a TAT-signal peptide, and therefore it stays in the cytoplasm (Dahl et al., 2013). This TAT-signal peptide also does not exist in the SoeABC-like protein of the cluster 2. Until now, no activity and substrate specificity have been proven for this second Soe-like cluster. Therefore, we propose that this Soe-like protein as a possible candidate for co-oxidation of As(III) and sulfite (SO_3_^2-^), or oxidation of thioarsenate. Moreover, we hypothesize that the observed oxidation has rather the aim of detoxifying the cell as both strains were unable to grow on As(III) as a sole electron donor (Supplementary Figure 2).

Multiple putative sulfurtransferases annotated as DsrE/F-like genes were found up- or downregulated in the presence of As(III). The sulfurtransferase DsrEFH binds to sulfur via a conserved cysteine of the DsrE and transports it to the DsrC in the reverse Dsr system of elemental sulfur oxidation to sulfite (Stockdreher et al., 2012). The function of a cysteine in an active site is known to be inactivated by the binding of As(III) to the sulfhydryl group (Shen et al., 2013). One possibility for their change in expression could therefore be either their induction to compensate for the inactivation (upregulation) or their reduction to shut down the pathway (downregulation).

### (5) Recombination and EnergyGeneration

Interestingly, genes for genetic recombination were downregulated. These include different transposases and integrases. This is in contradiction with the findings of Gualco et al. (2004) where a rise in the amount of recombinants in conjugation and transduction, and transposition of the Tn9 was observed when As(III) at a sub-MIC (Minimal Inhibitory Concentration) was added. This is in agreement with the general understanding that stressful environmental conditions induce genetic variation in bacteria via mutations and recombination (Bjedov et al., 2003; Foster, 2005, 2007; Prudhomme et al., 2006; Schuurmans et al., 2014).

In addition, genes are induced that are involved in the various pathways for the electron transfer in oxidative phosphorylation including the NADH:ubiquinone oxidoreductase in both strains, a cytochrome c synthesis gene and a Na^+^/H^+^ antiporter subunit in *Tv. thiocyanoxidans* ARh2^T^. The transcriptional upregulation of these complexes are commonly observed in bacteria in the presence of arsenic (Andres and Bertin, 2016). One explanation could be that arsenic works as an uncoupler of the membrane potential and as an alternative substrate of ATPase, which could impair NADH and ATP production. To ensuring adequate NADH and ATP production, *Tv. thiocyanoxidans* ARh2^T^ compensates this effect by the upregulation of the NADH:ubiquinone oxidoreductase, the Na^+^/H^+^ antiporter and by increased cytochrome c synthesis.

## Conclusion

In this study, we identified the putative potential of arsenic metabolism by the presence of Arx in 14 *Thioalkalivibrio* strains, and of Arr in two. Furthermore, we investigated the main mechanisms of arsenite resistance for *Tv. jannaschii* ALM2^T^ and *Tv. thiocyanoxidans* ARh2^T^. These strains do not share the same resistance to As(III), which is reflected in their growth response to different As(III) concentrations, in their repertoire of arsenic resistance genes, in their As(III)-oxidizing potential and in their transcriptome. From the gene expression, we discovered an involvement of vitamin B_12_ as the major player in the protection against arsenic-imposed oxidative stress, as well as the differential expression of DsrE/F-like proteins whose roles need to be elucidated in future research. Moreover, *Tv. jannaschii* ALM2^T^ induced the transcription of the *ars* gene operon and the Pst system, and *Tv. thiocanoxidans* ARh2^T^ increased expression of the *sox* and *apr* genes as well as different heat shock proteins. Comparing our results with the work of Fisher et al. (2008), we can postulate the formation of thioarsenates in the *Thioalkalivibrio* cultures, which were then microbiologically further oxidized by an as yet unknown enzymatic pathway to As(V). We hypothesize that a Soe-like protein is responsible for this oxidation, but evidence must be obtained by future work.

## Data Availability

The datasets generated for this study are deposited in the NCBI Sequence Read Archive under SRA accession numbers SRX5567239 to SRX5567253.

## Author Contributions

A-CA carried out the cultivation, the comparative sequence analyses, the RNA-Seq data analysis by sleuth, and drafted the manuscript. LC and MC carried out the analysis of the arsenic species. GM carried out the RNA-Seq data analysis by CLC. JS, DS, and GM assisted in the interpretation of the results, and together with LC provided a critical review of the manuscript. All authors read and approved the final version of the manuscript.

## Conflict of Interest Statement

The authors declare that the research was conducted in the absence of any commercial or financial relationships that could be construed as a potential conflict of interest.
