# Supplementary material for: Transcriptomic Analysis of Two Thioalkalivibrio Species Under Arsenite Stress Revealed a Potential Candidate Gene for an Alternative Arsenite Oxidation Pathway
Source: Front Microbiol. 2019 Jul 4;10:1514. doi: 10.3389/fmicb.2019.01514 (PMC6620896; doi:10.3389/fmicb.2019.01514)
Supplement: Supplementary file 1 [file Data_Sheet_1.docx]

Supplementary Material

Transcriptomic analysis of two Thioalkalivibrio species under arsenite stress revealed a potential candidate gene for an alternative arsenite oxidation pathway

Anne-Catherine Ahn, Lucia Cavalca, Milena Colombo, J. Merijn Schuurmans, Dimitry Y. Sorokin, Gerard Muyzer^*^

*** Correspondence:** Prof. Dr. Gerard Muyzer: g.muijzer@uva.nl

**
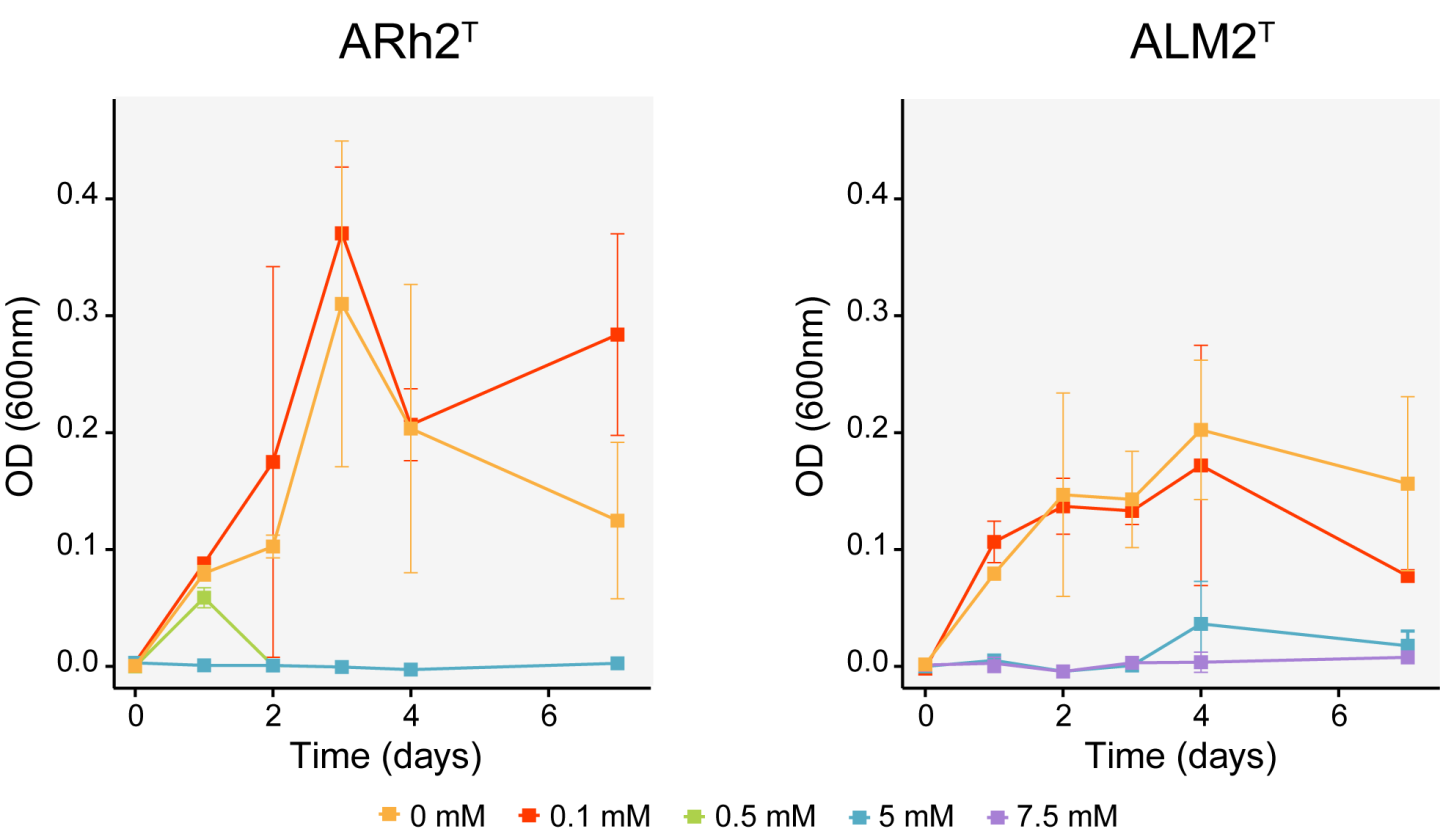
**

Supplementary Figure 1. Growth curves of *Tv. thiocyanoxidans* ARh2^T^ and *Tv. jannaschii* ALM2^T^ with various As(III) concentrations. Cultures were grown aerobically and with thiosulfate as their electron donor.


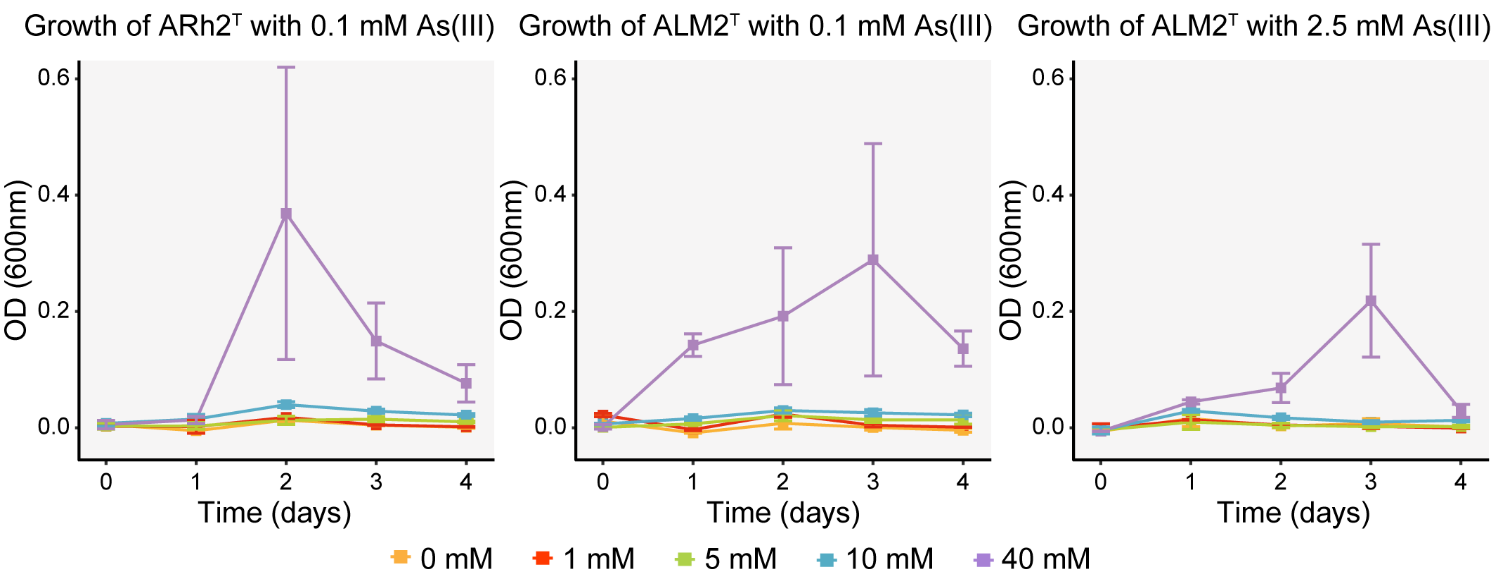


Supplementary Figure 2. Growth curves of *Tv. thiocyanoxidans* ARh2^T^ and *Tv. jannaschii* ALM2^T^ with various thiosulfate concentrations. Cultures were grown aerobically and with As(III).

**
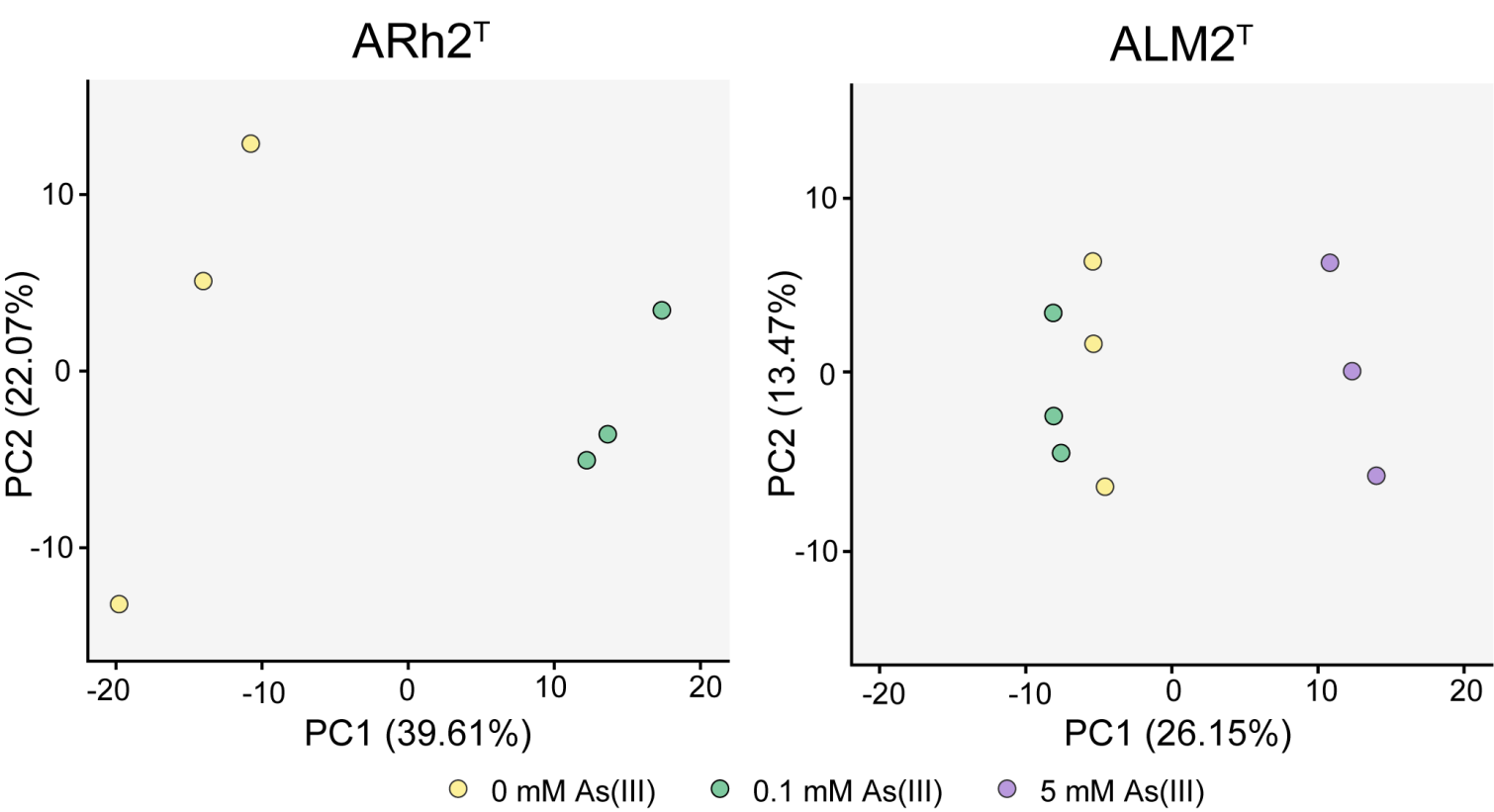
**

**Supplementary Figure 3**. Principle component analysis for the log_2_ RNA-Seq data of (A) *Tv. thiocyanoxidans* ARh2^T^ and (B) *Tv. jannaschii* ALM2^T^.

**
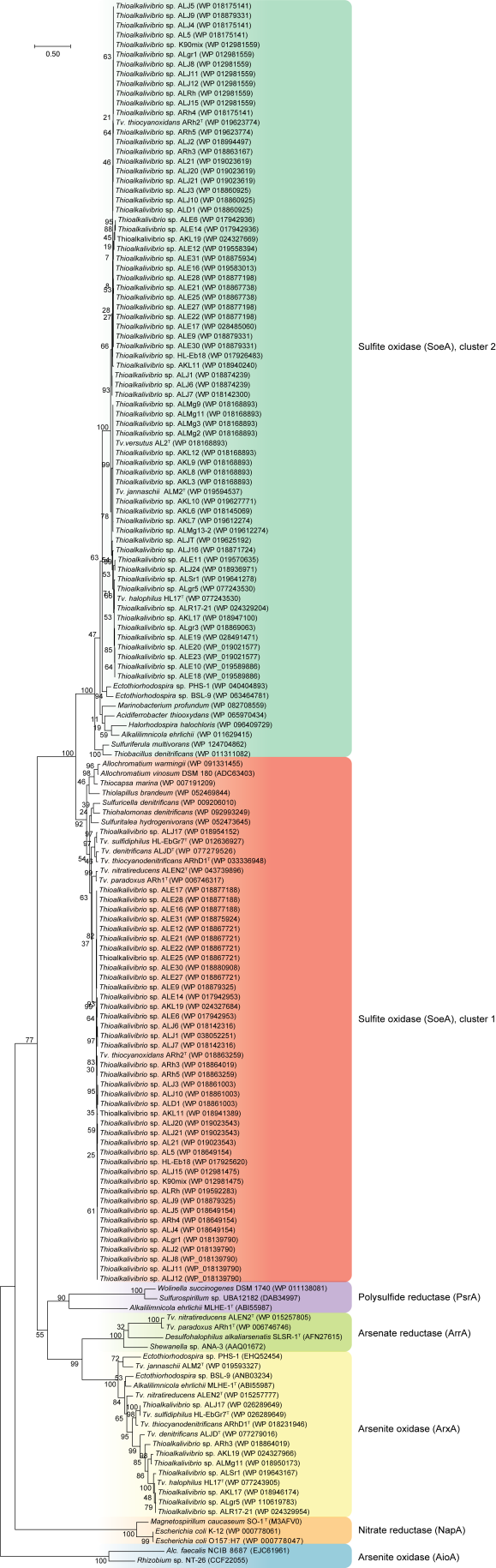
**

Supplementary Figure 4. Phylogenetic tree built with amino acid sequences of SoeA, SoeA-like and reference molybterin oxidoreductases. Accession number is provided for each sequence in the figure.

**Supplementary Table 1.** Differential expression data calculated with sleuth for the RNA-Seq samples of *Tv. thiocyanoxidans* ARh2^T^ (0.1 mM/0 mM As(III)), *Tv. jannaschii* ALM2^T^ (0.1 mM/0 mM As(III)) and *Tv. jannaschii* ALM2^T^ (5 mM/0 mM As(III)). The tables include the locus tag, *P*-value, q-value, b-value (beta-value), ste of b-value (standard error of the beta-value), mean of obs (mean of natural log counts of observations), var of obs (variance of observation), tech var (technical variance of observation from the bootstraps), sigma sq (raw estimator of the variance once the technical variance has been removed), smooth sigma sq (smooth regression fit for the shrinkage estimation), final sigma sq (max(sigma sq, smooth sigma sq); used for covariance estimation of beta), protein product and the raw read counts for the different RNA-Seq samples.

**Supplementary Table 2.** Differential expression data calculated with CLC for the RNA-Seq samples of *Tv. thiocyanoxidans* ARh2^T^ (0.1 mM/0 mM As(III)), *Tv. jannaschii* ALM2^T^ (0.1 mM/0 mM As(III) and 5 mM/0 mM As(III)). The tables include the locus tag, max group mean, log_2_ fold change, fold change, *P*-value, FDR *P*-value, Bonferroni and protein product.

**Supplementary Table 3**. Locus tags of genes used in the genomic comparison of arsenic resistance genes in *Alkalilimnicola ehrlichii* MLHE-1^T^, *Tv. jannaschii* ALM2^T^ and *Tv. thiocyanoxidans* ARh2^T^ (Figure 1).

**Supplementary Table 4.** List of putative ArsR proteins in *Tv. jannaschii* ALM2^T^ and *Tv. thiocyanoxidans* ARh2^T^ discovered by BLASTp using as subject the ArsR of *A. ehrlichii* MLHE-1^T^ (Mlg_2713) and of *Tv. jannaschii* ALM2^T^ (F816_RS0102085).

**Supplementary Table 5.** General information on the individual RNA-Seq samples analyzed by sleuth.

**Supplementary Table 6.** Most up- and downregulated genes in *Tv. thiocyanoxidans* ARh2^T^ during growth with 0.1 mM As(III) and compared to growth with 0 mM As(III).

**Supplementary Table 7.** Most up- and downregulated genes in *Tv. jannaschii* ALM2^T^ during growth with 0.1 mM As(III) and compared to growth with 0 mM As(III).

**Supplementary Table 8.** Most up- and downregulated genes for *Tv. jannaschii* ALM2^T^ during growth with 5 mM As(III) and compared to growth with 0 mM As(III).

**Supplementary Table 9.** Locus tags and differential expression values of genes used in the conceptual model.
